# Evaluation of the association between serum danger molecules at diagnosis and survival outcomes in patients with diffuse large B cell lymphoma

**DOI:** 10.3892/mi.2025.224

**Published:** 2025-03-05

**Authors:** Rafiye Çiftçiler, Ali Erdinc Ciftciler, Cem Selim

**Affiliations:** 1 Division of Hematology, Department of Internal Medicine, Faculty of Medicine, Selçuk University, 42131 Konya, Turkey; 2 Department of General Surgery, Konya Numune Hospital, 42060 Konya, Turkey

**Keywords:** diffuse large B cell lymphoma, damage-associated molecular patterns, fibrinogen, uric acid

## Abstract

When cellular stress or tissue injury occurs, molecules known as damage-associated molecular patterns are generated. The aim of the present was to examine the association between serum danger molecules and survival outcomes in patients with diffuse large B cell lymphoma (DLBCL) at the time of diagnosis. The present study was carried out retrospectively. An evaluation was conducted on 122 patients with DLBCL who were diagnosed at a tertiary care center between 2011 and 2024. The laboratory results of the patients diagnosed with DLBCL, which were examined in detail at diagnosis, were examined retrospectively. The median age of the patients was 59 years (range, 22-87 years) and 56.6% (n=69) of the patients were female. The 5-year overall survival (OS) rates of the patients who had a fibrinogen level ≤424 mg/dl and of those who had a fibrinogen level >424 mg/dl at the time of diagnosis were 82 and 41%, respectively (P<0.001). The 5-year progression-free survival (PFS) rates of the patients with a fibrinogen level ≤424 mg/dl and of those with a fibrinogen level >424 mg/dl at the time of diagnosis were 91 and 63%, respectively (P=0.002). The 5-year OS rates of patients with a uric acid level ≤5 mg/dl and those with a uric acid level >5 mg/dl at the time of diagnosis were 74 and 44%, respectively (P=0.008). The 5-year PFS rates of patients with a uric acid level ≤5 mg/dl and those with a uric acid level >5 mg/dl at the time of diagnosis were 83 and 71%, respectively (P=0.95). On the whole, the present study demonstrates that high uric acid and fibrinogen levels are negative prognostic factors for the OS and PFS of patients with DLBCL. These data may indicate that uric acid and fibrinogen levels at the time of diagnosis may provide predictive information to the existing library of clinical risk prediction tools in DLBCL.

## Introduction

Cancer cells must create mechanisms with which to evade the host immune system to manifest as neoplastic entities fully. This process, also known as ‘immune evasion’, is a characteristic of malignancies. Cancer immune escape, initially described in solid tumors, is a feature of several hematological malignancies (1). When cellular stress or tissue injury occurs, molecules known as damage-associated molecular patterns (DAMPs) are generated. These molecules are known as endogenous danger signals as they trigger potent inflammatory reactions by triggering the innate immune system during non-infectious inflammation (2-4). DAMPs are molecules generated upon cellular stress or tissue injury (3,4). Previous research has demonstrated that DAMPs, by causing inflammation, play a key role in the pathophysiology of human disorders (5). DAMPs include fibrinogen and uric acid. By activating the NOD-like receptor protein 3, uric acid encourages T-cells to release interleukin (IL)-1β, while fibrinogen stimulates macrophages to release inflammatory cytokines through Toll-like receptor (TLR)2 or 4 (2,6).

Although DAMPs contribute to the defense system of the host, they promote pathological inflammatory responses. The well-known promoters of carcinogenesis include IL-1, IL-6 and lymphotoxin (LT)-β. DAMPs produce IL-1, IL-6, LT-β, IFN-γ, TNF and transforming growth factor (TGF)-β when they activate inflammatory pathways (7,8). Examples of these DAMPs include high-mobility group box 1 (HMGB1), S100 proteins and heat-shock proteins (HSPs). Adenosine, uric acid, ATP, IL-1α and immunosuppression all contribute to carcinogenesis through angiogenesis, inflammation and tumor cell proliferation. In this instance, it appears that DAMPs promote the growth of tumors during the initial phases of carcinogenesis (9). Fibrinogen and uric acid, two DAMPs that are assessed and suitable therapeutic targets, were selected for analysis in the present study. The aim of the present study was to examine the association between serum danger molecules and the survival outcomes of patients with diffuse large B cell lymphoma (DLBCL) at the time of diagnosis.

## Patients and methods

### Study design and data collection

The present study was carried out retrospectively. An evaluation was conducted on 122 patients with DLBCL who were diagnosed at Selcuk University Hospital (Konya, Turkey) between 2011 and 2024. The hospital database provided the demographic information of the patients. As per the Declaration of Helsinki 1964, all ethical issues were rigorously adhered to. It was determined from the patient records that all the study subjects had provided informed consent at the time of hospitalization, prior to the administration of chemotherapy, and before receiving any other pertinent diagnostic or therapeutic standard of care, by the Selcuk Medical School's standard of care. Ethics committee approval was received from the Selçuk University Faculty of Medicine (dated October 22, 2024; no. 2024/533). The laboratory results of the patients diagnosed with DLBCL, which were examined in detail at the time of diagnosis, were examined retrospectively. The inclusion criteria for the study were that the patients were >18 years of age, had a diagnosis of DLBCL, and the laboratory parameters analyzed during diagnosis could be obtained from the hospital database. Patients whose data could not be accessed were excluded from the study.

### Patients, disease characteristics and definitions

Between 2011 and 2024, 122 patients with DLBCL who received care in the Hematology Clinic of Selçuk University were assessed. The study participants were adults who had just been diagnosed with DLBCL and were being treated in the Department of Hematology of Selçuk University. All patients had a pathological diagnosis. Bone marrow aspiration and biopsy were performed for staging purposes in each patient diagnosed with DLBCL. Additionally, each patient underwent positron emission tomography for staging purposes.

High and low fibrinogen and uric acid levels were determined according to the median fibrinogen and uric acid levels of all patients. Patients with fibrinogen levels >424 mg/dl at the time of diagnosis were evaluated as patients with high fibrinogen levels, while patients with fibrinogen levels ≤424 mg/dl were evaluated as patients with low fibrinogen levels. Patients with uric acid levels >5 mg/dl at the time of diagnosis were evaluated as patients with high uric acid levels, while patients with uric acid levels ≤5 mg/dl were evaluated as those with low uric acid levels.

Overall survival (OS) and progression-free survival (PFS) were used to evaluate the survival outcomes of the patients. OS was evaluated as the time during which patients were still alive after receiving the diagnosis of DLBCL. PFS was evaluated as the period during which the disease did not worsen or progress in patients with DLBCL during and after treatment.

### Statistical analyses

The statistical analyses utilized IBM Corp.'s SPSS 25 (IBM Corp.). Both analytical and visual approaches (such as probability maps and histograms) were used to explore the variables. The Kolmogorov-Smirnov/Shapiro-Wilk test was utilized to confirm the normality of the data. Chi-squared tests were used to perform statistical comparisons of categorical data. The Student's t-test was employed to compare continuous numerical data between two independent samples. Kaplan-Meier analysis was employed to conduct survival analyses. The log rank test was used to determine significance. Cox regression analysis was used to provide a multivariate study of survival factors. The multivariate analysis comprised parameters that exhibited P≤0.15 in the univariate analysis. A value of P<0.05 was considered to indicate a statistically significant difference.

## Results

### Patients and disease characteristics

A total of 122 patients were enrolled in the present study between 2011 and 2024. The median age of the patients was 59 years (range, 22-87 years) and 56.6% (n=69) of the patients were female. All patients (100%) were diagnosed with DLBCL. The median fibrinogen level was 424 mg/dl (range, 205-1,165 mg/dl) and the median uric acid level was 5.0 mg/dl (range, 1.5-12.9 mg/dl) at the time of diagnosis. In total, 53 patients (43.4%) were evaluated as ECOG PS 1. According to the Ann Arbor staging system, 64 patients (52.5%) were evaluated as stage IV according to the PET CT and bone marrow biopsy results. Extranodal involvement was present in 84 patients (68.9%) at the time of diagnosis. The laboratory and clinical characteristics of the patients are summarized in Table I.

### Laboratory and clinical characteristics of the patients according to danger molecule levels

In the patients with high fibrinogen levels at the time of diagnosis, age, neutrophil counts, platelet counts, sedimentation, C-reactive protein (CRP) levels, ECOG PS, disease stage, International Prognostic Index (IPI), Revised-International Prognostic Index (R-IPI), and National Comprehensive Cancer Network-International Prognostic Index (NCCN-IPI) scores were higher. By contrast, the albumin levels were significantly lower in patients with high fibrinogen levels. The relapse and mortality rates were also higher in patients with high fibrinogen levels (Table II).

In patients with high uric acid levels at the time of diagnosis, age and lactate dehydrogenase (LDH) levels were higher, while B12 levels were significantly lower than in patients with low uric acid levels. The mortality rates were also higher in patients with high uric acid levels (Table III).

### Overall outcomes

**Figure 1:**
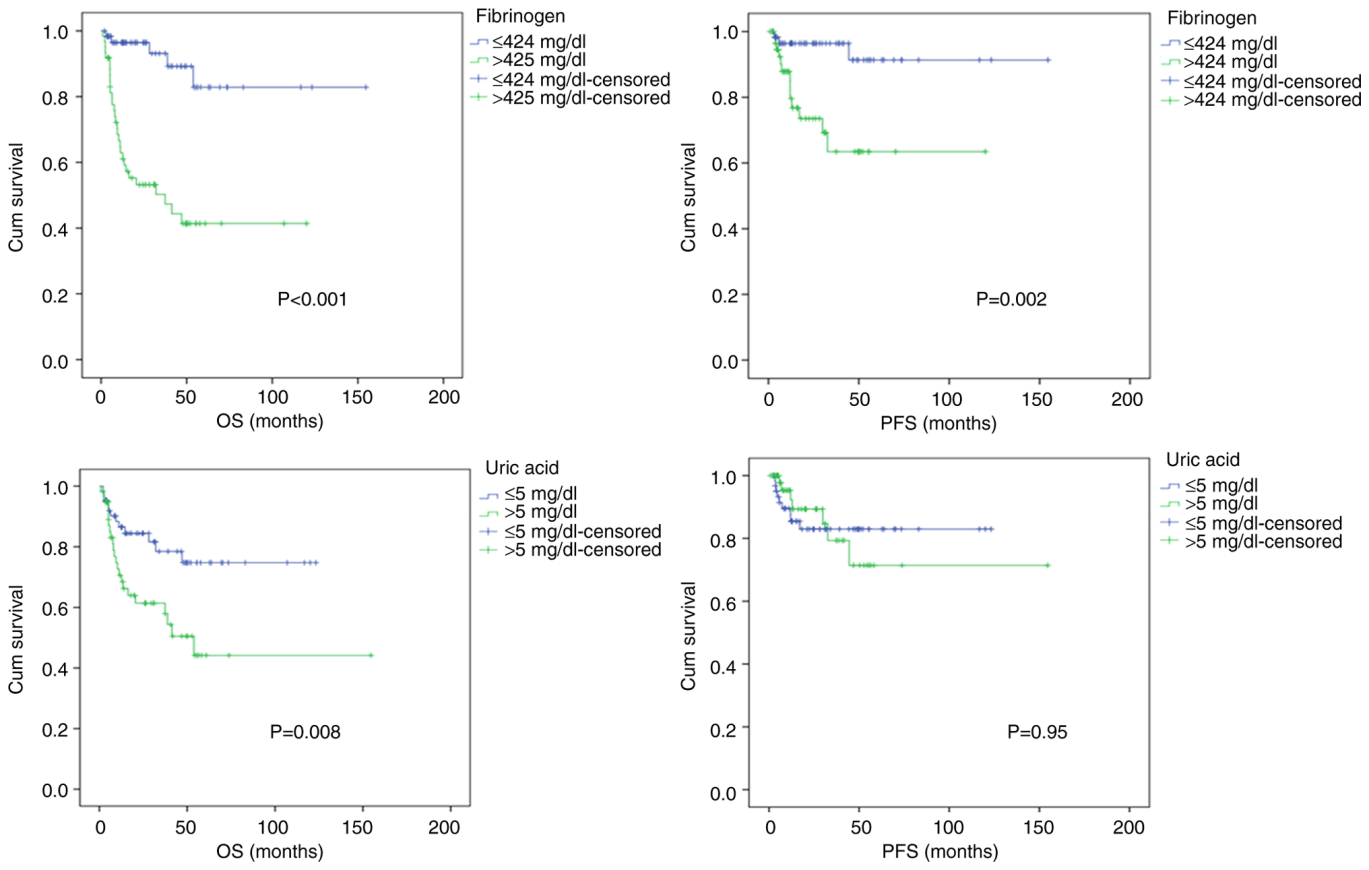
Overall survival and progression-free survival according to fibrinogen and uric acid levels at the time of diagnosis.

The median OS rate was 20 months (range, 0.8-154.5 months) for all patients. Of the 122 patients included in the study, 61 patients (50%) had fibrinogen levels ≤424 at the time of diagnosis. The other 61 patients (50%) had fibrinogen levels >424. The 5-year OS rates of the patients who had a fibrinogen level ≤424 mg/dl and of those who had a fibrinogen level >424 mg/dl at the time of diagnosis were 82 and 41%, respectively (P<0.001). The 5-year PFS rates of the patients who had a fibrinogen level ≤424 mg/dl and of those who had a fibrinogen level >424 mg/dl at the time of diagnosis were 91 and 63%, respectively (P=0.002) (Fig. 1).

When the patients were evaluated in terms of uric acid, another danger molecule, 64 patients (52.5%) had uric acid levels ≤5 mg/dl, while 58 patients (47.5%) had uric acid levels >5 mg/dl at the time of diagnosis. The 5-year OS rates of the patients with a uric acid level ≤5 mg/dl and those with a uric acid level >5 mg/dl at the time of diagnosis were 74 and 44%, respectively (P=0.008). The 5-year PFS rates of the patients who had a uric acid level ≤5 mg/dl those who had a uric acid level >5 mg/dl at the time of diagnosis were 83 and 71%, respectively (P=0.95) (Fig. 1).

### Cox regression analyses

In univariate analyses, the variables that affected OS were determined to be an age ≤58 years (P=0.001), fibrinogen levels ≤424 mg/dl (P<0.001), uric acid levels ≤5 mg/dl) (P=0.008), an ECOG performance score of 0, 1, 2 and 3) (P<0.001), a disease stage of I, II, III and IV) (P=0.01), an International Prognostic Score of low, low-intermediate, high-intermediate, high) (P<0.001), a revised IPI of very good/good/poor (P<0.001), NCCN IPI of low, low-intermediate, high-intermediate, high (P<0.001), extranodal involvement (P=0.14) and treatment regimen (P=0.12). Cox regression analysis revealed that fibrinogen levels ≤424 mg/dl) (P=0.002), a revised IPI (very good/good/poor; P=0.03) and treatment regimen (R-CHOP; P=0.04) were the parameters predicting OS (Table IV).

In the univariate analyses, the variables that affected PFS were determined to be an age ≤58 years (P=0.08), the male sex (P=0.03), fibrinogen levels ≤424 mg/dl (P=0.002), an ECOG performance score (0, 1, 2, 3; P=0.01), disease stage (I, II, III, IV; P=0.07), an International Prognostic Score (low, low-intermediate, high-intermediate, high; P<0.001), a revised IPI (very good/good/poor; P=0.02) and NCCN IPI (low, low-intermediate, high-intermediate, high; P=0.003). Cox regression analysis revealed only the fibrinogen level (≤424 mg/dl) (P=0.02), as a parameter predicting PFS (Table IV).

## Discussion

Numerous DAMPs have been found since Matzinger (10) developed the hazard model, and the number of DAMPs is continually increasing (4,5,10,11). Following tissue damage or cell death, DAMPs are released from the intracellular or extracellular area (11). Macrophages can identify these DAMPs, and various pathways, such as TLRs and inflammasomes, can cause an inflammatory response (11,12). DAMPs can come from a variety of sources and include plasma proteins such as fibrinogen, Gc-globulin and serum amyloid A, as well as extracellular proteins such as biglycan and tenascin C and intracellular proteins, such as HMGB1, histones, S100 proteins and HSPs (11,13,14).

The role and mechanisms of DAMPs in the etiology of cancer remain a matter of debate. By causing persistent inflammation, which is a complex risk factor for tumor growth, DAMPs may influence tumor progression (7,9). To the best of our understanding, well-known promoters of carcinogenesis include IL-1, IL-6 and LT-β (7-9). DAMPs that trigger inflammatory pathways and produce IL-1, IL-6, LT-β, IFN-γ, TNF and TGF-β include HMGB1, S100 proteins and HSPs (9). Through inflammation, immunosuppression, angiogenesis and tumor cell proliferation, ATP, IL-1α, adenosine and uric acid also contribute to carcinogenesis (9). According to this, DAMPs appear to promote the growth of tumors during the early phases of carcinogenesis (9).

Plasma glycoproteins, such as fibrinogen are mostly produced by liver cells, although they are also produced by tumor and epithelial cells (15). Fibrinogen is physiologically focused on wound healing, coagulation and platelet aggregation (16). Fibrinogen is one of the positive acute phase proteins, known for their increased quantities during inflammation (17). Shehata *et al* (18) revealed an association between a number of clinical and laboratory traits of patients with DLBCL and hyperfibrinogenemia. Additionally, they evaluated the potential significance of hyperfibrinogenemia in the prognoses of these individuals (18). According to their study, there was a strong association between the baseline fibrinogen level and laboratory markers, such as platelet count, serum LDH levels, B2-microglobulin and red cell distribution width. There were no statistically significant associations observed between baseline fibrinogen levels and OS, PFS, or responsiveness to therapy (18). In another study, in patients with non-Hodgkin lymphoma, Wang *et al* (19) found a strong association between the number of extranodal locations implicated and hyperfibrinogenemia. Additionally, they discovered a propensity for patients with DLBCL with baseline hyperfibrinogenemia to have a lower 2-PFS (19). Troppan *et al* (20) revealed that a high fibrinogen plasma level was associated with decreased 5-year OS and a 5-year PFS in patients with DLBCL (P<0.001). Furthermore, in their study, in a multivariate analysis, elevated serum fibrinogen levels were found to be an independent marker of poor clinical outcomes: 5-year OS [hazard ratio (HR), 1.69; 95% confidence interval (CI), 1.06-2.72, P=0.029] and 5-year PFS (HR, 1.68; 95% CI, 1.08-2.61, P=0.021) (20).

On the other hand, the ultimate byproduct of purine metabolism, uric acid, demonstrates a high nucleic acid turnover rate in cellular components. Consequently, elevated uric acid levels in the blood are frequently observed in individuals with solid tumors or hematological malignancies. Crucially, multiple studies have shown that high uric acid levels are associated with a poor prognosis of patients with several solid cancer types, such as pancreatic, esophageal and colon cancer, as well as an increased risk of cancer incidence and mortality (21-23). Prochazka *et al* (24) demonstrated that patients with DLBCL with elevated uric acid levels before therapy had a statistically significant and clinically significant low OS and PFS. To date, only a limited number of studies have examined the association between pre-treatment uric acid levels and disease prognosis in patients with DLBCL. Graft vs. host disease (GvHD) and allogeneic hematopoietic stem cell transplantation (allo-HCT) cause injured tissue to emit fibrinogen and uric acid (25). Previously, Çelik *et al* (25) assessed the effects of fibrinogen levels and uric acid on GvHD survival. The levels of uric acid and fibrinogen were assessed on day 0 of allo-HCT and on days 0, 7, 14 and 28 of GvHD. In multivariable models, fibrinogen GvHD day 0 was the independent predictor of OS, PFS, and non-relapse mortality (NRM). Additionally, OS and NRM were independently predicted by uric acid GvHD on day 28. According to that study, a considerably shorter OS and greater NRM were linked to hypouricemia and hypofibrinogenemia (25).

The present study evaluated the effects of fibrinogen and uric acid levels, which are danger molecules at the time of diagnosis, on survival outcomes in 122 patients with DLBCL. Patients with higher fibrinogen levels at the time of diagnosis were older, and these patients also had higher neutrophil counts, platelet counts, sedimentation rates and CRP levels at the time of diagnosis, while the albumin levels were lower. When evaluated clinically, the ECOG performance score, disease stage, International Prognostic Score, revised IPI, NCCN IPI, relapse rate and mortality rate were higher in patients with high fibrinogen levels. Patients with higher uric acid levels at the time of diagnosis were also older, and these patients also had higher LDH levels and lower vitamin B12 levels at diagnosis. When evaluated clinically, only the mortality rate was higher in patients with high uric acid levels, unlike the fibrinogen level. OS and PFS were found to be statistically significantly higher in patients with lower fibrinogen levels at the time of diagnosis. OS was statistically significantly higher in patients with lower uric acid levels at the time of diagnosis, while no statistically significant difference was observed in PFS. When evaluated with multivariate analysis, lower fibrinogen levels at the time of diagnosis, lower R-IPS scores, and chemotherapy protocol (R-CHOP) were found to have improved OS rates. In the multivariate analysis, only lower fibrinogen levels at diagnosis were associated with better PFS rates.

The present study had some limitations. Only fibrinogen and uric acid levels were evaluated among the danger molecules. Since this was a retrospective study, other danger molecules could not be evaluated. However, since fibrinogen and uric acid are easily accessible tests and can be studied in almost every patient, it is deemed that this study may prove to be useful for clinical practice.

In conclusion, the present study demonstrated that high uric acid and fibrinogen levels were negative prognostic factors for OS and PFS in patients with DLBCL. These data may indicate that uric acid and fibrinogen levels at the time of diagnosis may provide predictive information to the existing library of clinical risk prediction tools in DLBCL.

## Figures and Tables

**Table I:** Laboratory and clinical characteristics of all the patients in the present study.

Parameter	Value; n (%) or median (range)
Age (median years, range)	58 (22-87)
Sex (female/male)	69 (56.6)/53 (43.4)
Hemoglobin (mg/dl)	12.4 (5.7-17.9)
Leucocyte (x10^9^/l)	7.4 (1.2-77.2)
Neutrophil (x10^9^/l)	5.1 (0.9-20.0)
Monocytes (x10^9^/l)	1.4 (0.1-16.1)
Lymphocytes (x10^9^/l)	0.6 (0.08-46.4)
Platelets (x10^9^/l)	263 (42-691)
Sedimentation rate (mm/h)	23 (2-120)
C reactive protein (mg/l)	20.5 (2.1-396)
Ferritin (ng/m/l)	192.4 (5.8-15,000)
Fibrinogen (mg/dl)	424 (205-1,165)
Uric acid (mg/dl)	5.0 (1.5-12.9)
Albumin (g/dl)	3.8 (2.2-4.9)
Lactate dehydrogenase (U/l)	291 (122-4,065)
B12 (pg/ml)	308 (75-1,965)
ECOG performance score (0/1/2/3)	30 (24.6)/53 (43.4)/24 (19.7)/15 (12.3)
Stage (I/II/III/IV)	11 (9.0)/26 (21.3)/21 (17.2)/64 (52.5)
International Prognostic Score (low/low-intermediate/high-intermediate/high)	35 (28.7)/39 (32.0)/26 (21.3)/22 (18.0)
Revised IPI (very good/good/poor)	5 (4.1)/71 (58.2)/46 (37.7)
NCCN IPI (low/low-intermediate/high-intermediate/high)	10 (8.2)/68 (55.7)/32 (26.2)/12 (9.8)
Extranodal involvement	84 (68.9%)

ECOG, Eastern Cooperative Oncology Group; revised IPI, revised International Prognostic Index; NCCN IPI, National Comprehensive Cancer Network International Prognostic Index.

**Table II:** Laboratory and clinical characteristics according to the fibrinogen level at diagnosis.

Parameter	Patients with a fibrinogen level ≤424 mg/dl at diagnosis; n, (%), median (range)	Patients with a fibrinogen level >424 mg/dl at diagnosis; n, (%), median (range)	P-value
No. of patients (%)	61 (50%)	61 (50%)	
Age in years. median (range)	53 (22-85)	61 (22-87)	0.003
Sex (female/male)	33 (47.8)/28 (52.8)	36 (52.2)/25 (47.2)	0.58
Hemoglobin (mg/dl)	12.7 (5.7-17.9)	12.1 (7.0-15.7)	0.06
Leucocytes (x10^9^/l)	6.5 (4.2-20.5)	8.3 (1.2-77.2)	0.08
Neutrophils (x10^9^/l)	4.5 (1.7-18.6)	5.8 (0.9-20.0)	0.03
Monocytes (x10^9^/l)	0.5 (0.08-13.0)	0.7 (0.1-46.4)	0.36
Lymphocytes (x10^9^/l)	1.4 (0.2-5.3)	1.5 (0.1-16.1)	0.47
Platelets (x10^9^/l)	238 (42-496)	267 (102-691)	0.04
Sedimentation rate (mm/h)	18 (2-78)	29 (4-120)	<0.001
C reactive protein (mg/l)	10 (2.1-396)	34.7 (2.9-245)	0.03
Ferritin (ng/m/l)	110 (12.6-2,000)	214 (5.8-14,000)	0.24
Uric acid (mg/dl)	5 (2.3-10.0)	5.2 (1.5-12.9)	0.41
Albumin (g/dl)	4.1 (2.4-4.9)	3.7 (2.2-4.7)	0.003
Lactate dehydrogenase (U/l)	267 (153-1,980)	314 (122-4,065)	0.11
B12 (pg/ml)	294 (75-727)	312 (158-1,965)	0.08
ECOG performance score (0/1/2/3)	15 (24.6)/33 (54.1)/9 (14.8)/4 (6.6)	15 (24.6)/20 (32.8)/15 (24.6)/11 (18.0)	0.04
Stage (I/II/III/IV)	6 (9.8)/21 (34.4)/12 (19.7)/22 (36.1)	5 (8.2)/5 (8.2)/9 (14.8)/42 (68.9)	0.001
International prognostic score (low/low-intermediate/high-intermediate/high)	25 (41.0)/23 (37.7)/8 (13.1)/5 (8.2)	10 (16.4)/16 (26.2)/18 (29.5)/17 (27.9)	<0.001
Revised IPI (very good/good/poor)	20 (32.8)/32(52.5)/9 (14.8)	8 (13.1)/29(47.5)/24 (39.3)	0.002
NCCN IPI (low/low-intermediate/high-intermediate/high)	7 (11.5)/20 (32.8)/19 (31.1)/15 (24.6)	3 (4.9)/10 (16.4)/19 (31.1)/29 (47.5)	0.023
Extranodal involvement, yes/no	37 (60.7)/24 (39.3)	47 (77.0)/14 (23%)	0.05
Relapse rate, yes/no	3 (4.9)/58 (95.1)	13 (21.3)/48 (78.7)	0.007
Mortality rate, yes/no	5 (8.2)/56 (91.8)	30 (49.2)/31 (50.8)	<0.001

ECOG, Eastern Cooperative Oncology Group; revised IPI, revised International Prognostic Index; NCCN IPI, National Comprehensive Cancer Network International Prognostic Index.

**Table III:** Laboratory and clinical characteristics according to uric acid level at diagnosis.

Parameter	Patients with uric acid ≤5 mg/dl at diagnosis; n, (%), median (range)	Patients with uric acid >5 mg/dl at diagnosis; n, (%), median (range)	P-value
No. of patients (%)	64 (52.4%)	58 (47.6%)	
Age in years, median (range)	54 (22-86)	60 (32-87)	0.03
Sex (female/male)	33 (51.6)/31 (48.4)	36 (62.1)/22 (37.9)	0.24
Hemoglobin (mg/dl)	12.3 (817-15.7)	12.4 (5.7-15.9)	0.65
Leucocytes (x10^9^/l)	7.4 (3.6-23.6)	7.4 (1.2-77.2)	0.40
Neutrophils (x10^9^/l)	5.1 (1.7-20.0)	5.0 (0.9-15.3)	0.62
Monocytes (x10^9^/l)	0.6 (0.08-1.6)	0.67 (0.10-46.4)	0.17
Lymphocytes (x10^9^/l)	1.4 (5.3-0.26)	1.4 (0.10-16.1)	0.26
Platelets (x10^9^/l)	266 (24-664)	233 (70-691)	0.43
Sedimentation rate (mm/h)	21.5 (2-120)	24 (2-97)	0.93
C reactive protein (mg/l)	15.9 (2.1-245)	23 (2.5-396)	0.77
Ferritin (ng/m/l)	199 (5.8-2,000)	169 (17-15,000)	0.57
Albümin (g/dl)	3.8 (2.2-4.9)	3.9 (2.2-4.7)	0.83
Lactate dehydrogenase (U/l)	254 (122-1,980)	381 (172-4,065)	0.007
B12 (pg/ml)	308 (75-1,965)	305 (140-652)	0.02
ECOG performance score (0/1/2/3)	20 (31.3)/28 (43.8)/11 (17.2)/5 (7.8)	10 (17.2)/25 (43.1)/13 (22.4)/10 (17.2)	0.16
Stage (I/II/III/IV)	7 (10.9)/15 (23.4)/12 (18.8)/30 (46.9)	4 (6.9)/11 (19.0)/9 (15.5)/34 (58.6)	0.61
International prognostic score (low/low-intermediate/high-intermediate/high)	21 (32.8)/23 (35.9)/13 (20.3)/7 (10.9)	14 (24.1)/16 (27.6)/13 (22.4)/15 (25.9)	0.15
Revised IPI (very good/good/poor)	16 (25.0)/35 (54.7)/13 (20.3)	12 (20.7)/26 (44.8)/20 (34.5)	0.21
NCCN IPI (low/low-intermediate/high-intermediate/high)	7 (10.9)/19 (29.7)/19 (29.7)/19 (29.7)	3 (5.2)/11 (19.0)/19 (32.8)/25 (43.1)	0.23
Extranodal involvement, yes/no	43 (67.2)/21 (32.8)	41 (70.7)/17 (29.3)	0.67
Relapse rate, yes/no	9 (14.1)/55 (85.9)	7 (12.1)/51 (57.9)	0.74
Mortality rate, yes/no	12 (18.8)/52 (71.2)	23 (39.7)/35 (60.3)	0.001

ECOG, Eastern Cooperative Oncology Group; revised IPI, revised International Prognostic Index; NCCN IPI, National Comprehensive Cancer Network International Prognostic Index.

**Table IV:** Univariate and multivariate analyses (Cox model) of overall survival and progression-free survival.

A, Parameters for overall survival					
			Multivariate analyses		
Parameter	Favorable factors	Univariate analyses P-value	Hazard ratio	95% confidence interval	P-value
Age	≤58 years	0.001	1.261	0.431-3.692	0.67
Sex		0.81	-	-	-
Fibrinogen level	≤424 mg/dl	<0.001	5.583	1.904-16.378	0.002
Uric acid level	≤5 mg/dl	0.008	1.688	0.771-3.696	0.19
ECOG performance score (0, 1, 2, 3)		<0.001	1.565	0.931-2.631	0.09
Stage (I, II, III, IV)		0.01	1.018	0.539-1.922	0.95
International Prognostic Score (low, low-intermediate, high-intermediate, high)		<0.001	0.979	0.434-2.207	0.95
Revised IPI (very good/good/poor)		<0.001	2.210	1.079-4.526	0.03
NCCN IPI (low, low-intermediate, high-intermediate, high)		<0.001	1.107	0.435-1.747	0.69
Extranodal involvement	None	0.14	1.107	0.452-2.711	0.82
Treatment regimen	R-CHOP	0.12	2.367	1.029-5.443	0.04
B, Parameters for progression-free survival					
			Multivariate analyses		
Parameter	Favorable factors	Univariate analyses P-value	Hazard ratio	95% confidence interval	P-value
Age	≤58 years	0.08	1.179	0.327-4.248	0.80
Gender	Male	0.03	3.529	0.945-13.176	0.06
Fibrinogen level	≤424 mg/dl	0.002	5.222	1.258-21.676	0.02
Uric acid level	≤5 mg/dl	0.95	-	-	-
ECOG performance score (0, 1, 2, 3)		0.01	1.798	0.778-4.155	0.17
Stage (I, II, III, IV)		0.07	1.394	0.648-3.003	0.39
International Prognostic Score (low, low-intermediate, high-intermediate, high)		<0.001	3.262	0.916-11.621	0.06
Revised IPI (very good/good/poor)		0.02	0.436	0.155-1.224	0.11
NCCN IPI (low, low-intermediate, high-intermediate, high)		0.003	0.800	0.314-2.042	0.64
Extranodal involvement	None	0.35	-	-	-
Treatment regimen	R-CHOP	0.54	-	-	-

ECOG, Eastern Cooperative Oncology Group; revised IPI, revised International Prognostic Index; NCCN IPI, National Comprehensive Cancer Network International Prognostic Index.

## Data Availability

The data generated in the present study may be requested from the corresponding author.
